# Deciphering the Assembly Processes of the Key Ecological Assemblages of Microbial Communities in Thirteen Full-Scale Wastewater Treatment Plants

**DOI:** 10.1264/jsme2.ME18107

**Published:** 2019-04-16

**Authors:** Liyuan Hou, Anyi Hu, Shaohua Chen, Kaisong Zhang, Sandi Orlić, Azhar Rashid, Chang-Ping Yu

**Affiliations:** 1 CAS Key Laboratory of Urban Pollutant Conversion, Institute of Urban Environment, Chinese Academy of Sciences Xiamen 361021 P. R. China; 2 Department of Civil and Environmental Engineering, University of Missouri USA; 3 Ruđer Bošković Institute Bijeničkacesta 54, 10000 Zagreb Croatia; 4 Center of Excellence for Science and Technology–integration of Mediterranean region– STIM Bijeničkacesta 54, 10000, Zagreb Croatia; 5 Nuclear Institute for Food and Agriculture Tarnab, Peshawar Pakistan; 6 Graduate Institute of Environmental Engineering, National Taiwan University Taipei 106 Taiwan

**Keywords:** activated sludge, microbial community, community assembly, core and satellite, habitat generalist and specialist, amplicon sequencing

## Abstract

Limited information is currently available on the assembly processes (deterministic vs. stochastic) shaping the compositions of key microbial communities in activated sludge (AS). The relative importance of deterministic and stochastic processes for key bacterial and archaeal assemblages (*i.e*., core-satellite and habitat generalist-specialist) in AS from 13 wastewater treatment plants in China was investigated using 16S rDNA amplicon sequencing. The results obtained indicated 1,388 and 369 core operational taxonomic units (OTUs), 1,038 and 1,683 satellite OTUs, 255 and 48 habitat generalist OTUs, and 192 and 111 habitat specialist OTUs for *Bacteria* and *Archaea*, respectively. The proportions of shared OTUs between core and habitat specialist communities were similar to or higher than those between core and habitat generalist communities, suggesting a stronger inter-linkage between the former two groups. Deterministic processes, indicated by abundance-based *β*-null models, were responsible for shaping core communities, in which NH_4_-N, OrgC/OrgN, Cr, and Ni were the main controlling factors. In contrast, satellite communities were predominantly influenced by stochastic processes. Moreover, we found that deterministic and stochastic processes were mainly responsible for shaping the assembly of habitat specialists and generalists, respectively. However, the influence of deterministic factors on habitat specialists remains unclear. The present study provides novel insights into the assembly mechanisms of AS microbial communities.

Wastewater treatment plants (WWTPs) are engineered systems that rely on complex microbial communities to remove organic matter and nutrients from municipal and industrial wastewaters (15, 26). The activated sludge (AS) process represents the most widely used biotechnological process in WWTPs (45). Control over key ecological groups of microorganisms (*i.e*., core-satellite and habitat generalist-specialist) in AS is important for the stable and efficient performance of WWTPs (23, 24). Therefore, detailed investigations on the assembly mechanisms of key ecological groups will contribute to the development of effective strategies for “microbial resource management” in the AS process. However, the assembly mechanisms of microbial communities remain unclear and debatable in both natural and engineered ecosystems.

The assembly of microbial communities is suggested to be influenced by a combination of complex ecological processes (*i.e*., deterministic and stochastic) (37). Two ecological theories —deterministic and stochastic—have generally been proposed. The deterministic theory describes traditional niche-based processes, in which deterministic factors, such as habitat heterogeneity, environmental conditions, and bio-interactions, play a critical role in structuring microbial communities (6, 30, 62, 63). On the other hand, the stochastic theory assumes that all species are ecologically equivalent and only depict random or neutral processes (*e.g*., birth, death, colonization, immigration, and speciation) (27, 30). Previous studies demonstrated the dominance of stochastic processes in sites featured by more benign environments, whereas deterministic processes play a predominant role in harsher environments (8, 40, 63). However, deterministic and stochastic processes were also found to be jointly responsible for shaping microbial community assembly in diverse environments (27). Previous studies provided a theoretical foundation for the optimization of WWTPs by investigating the rules underlying the assembly of AS microbial communities (7, 44). The structures of AS microbial communities were recently shown to be mainly regulated by environmental or operational variables (*e.g*., temperature, dissolved oxygen (DO), chemical oxygen demand (COD), and nutrients) (12, 24, 35, 54), suggesting that the dynamics of the AS microbial population is strongly controlled by deterministic factors (1, 23, 60). In contrast, other studies demonstrated that stochastic processes (*e.g*., historical, spatial, and evolutionary factors) played a more important role in shaping the structure of AS microbial communities (8, 54, 60). However, most of these studies focused on the ecological processes of AS microbial communities at the community level and limited information is currently available on the assembly dynamics of some key ecological groups within communities.

Microbial species within communities may be partitioned into core and satellite or habitat generalist and specialist groups. The core-satellite concept is based on the frequency and density of microorganisms within a range of sites. The core group contains species that are widely distributed and locally abundant, while the satellite group presents species that occur in low abundance at limited locations (50). In contrast to the core-satellite concept, the habitat generalist-specialist concept reflects the nature of the environmental tolerance of microbial species (41, 48). Habitat generalists are distributed in a wide range of diverse sites, while habitat specialists are more restricted in sites and have narrow environmental tolerance (27, 41). Since core and habitat generalist taxa have some common characteristics (*e.g*., advantages in the utilization of widespread resources and broad tolerance to environmental conditions), there may be some degree of overlap between core and habitat generalist populations (20, 29). However, previous findings indicate that two opposite (*i.e*., deterministic and stochastic) ecological processes are mainly responsible for the assembly of bacterial core and habitat generalist populations, respectively (22, 25, 29, 41, 48). A number of studies have shown that bacterial habitat specialists are more susceptible to extinction than bacterial habitat generalists when habitat conditions change (27, 28, 53), while the occurrence of bacterial satellite taxa is dominated by random dispersal (3, 22, 29). The assembly processes of archaeal ecological groups in natural and man-made environments have been investigated in less detail than those of *Bacteria*. A previous study demonstrated that the archaeal core community was more divergent from the satellite community in coastal sediment habitats than those of *Bacteria* and *Eukarya* (22). Monard *et al*. (36) found that deterministic processes were mainly responsible for shaping microbial community compositions across a terrestrial-freshwater gradient, in which few archaeal generalists but many archaeal habitat specialists were detected. Furthermore, previous findings suggested that core taxa are ubiquitous and play an essential role in the functioning of AS (24, 45), and deterministic factors were found to be more likely to control the core AS community structure (15, 34). However, the role of the random dispersal or immigration of rare taxa in the assembly of AS communities needs to be considered (16). Although an in-depth understanding of the distribution of key ecological groups (*i.e*., core-satellite and habitat generalist-specialist) is the most crucial step in managing microbial resources for ecosystem services (21, 36, 42, 45), the assembly mechanisms of these key ecologically functional groups in AS have not yet been elucidated in detail.

In the present study, our primary objectives were to investigate the distribution and corresponding community assembly mechanisms for the key ecological groups (*i.e*., core-satellite and habitat generalist-specialist) in two life domains (*i.e*., *Bacteria* and *Archaea*) from the AS samples of 13 WWTPs in two cities (Chongqing and Xiamen) in China. We specifically proposed the following hypotheses: (i) a considerable overlap will be present between core and habitat generalist groups because both of these groups have the ability to utilize wide-spread resources and have broader environmental tolerance; (ii) core and satellite communities will have different assembly patterns, with the assembly of core and satellite species being structured by deterministic and stochastic processes, respectively; and (iii) the assembly of habitat generalists and specialists will be dominated by stochastic and deterministic processes, respectively. The confirmation of these hypotheses will enhance our understanding of the role of AS microbial communities in WWTPs. In the present study, data were analyzed by 16S rRNA genes amplicon high-throughput sequencing, the *β*-diversity null model test, and a multivariate statistical analysis.

## Materials and Methods

### Sample collection and environmental parameter measurements

AS samples were collected from 13 full-scale WWTPs in Chongqing (105°11′E–110°11′E, 28°10′N–32°13′N) and Xiamen (117°54′E–118°21′E, 24°24′N–24°54′N), China in February 2010 (Fig. S1). General information on these WWTPs is listed in Table S1. Twelve of these WWTPs employ different suspended growth systems (AS processes), while only one of the WWTPs uses a biofilm system (biological aerated filter) as the biological treatment process (Table S1). Triplicate AS samples were randomly collected from the final sedimentation tank, mixed and stored in sterile 50-mL conical tubes (BD Biosciences, Bedford, MA, USA), transported to the laboratory in dry ice, and then stored at −80°C until further analyses.

Mean monthly air temperature data during the sampling time were obtained from local meteorological agencies as a proxy of water temperature (Table S2). The pH of AS samples was measured by UB-7 Ultra Basic pH-detection (Denver Instruments, Arvada, CO, USA). Ammonium (NH_4_-N), nitrite (NO_2_-N), and nitrate (NO_3_- N) levels were measured by a colorimetric method using a Lachat QC8500 Flow Injection Autoanalyzer (Lachat Instruments, Loveland, CO, USA). Organic matter contents (organic carbon [OrgC], organic nitrogen [OrgN], and organic sulfur [OrgS]) were measured by a Vario Max CNS analyzer (Elementar, Hanau, Germany). The concentrations of the following heavy metals: chromium (Cr), nickel (Ni), copper (Cu), zinc (Zn), cadmium (Cd), lead (Pb), and silver (Ag), were measured by an Agilent 7500cx ICP-MS (Agilent Technologies, Santa Clara, CA, USA).

### DNA extraction and quantification of bacterial and archaeal 16S rRNA genes

Genomic DNA was extracted from approximately 0.3 g of each AS sample using the FastDNA SPIN Kit for Soil (Qbiogene-MP Biomedicals, Irvine, CA, USA) according to the manufacturer’s instructions. In the quantification of bacterial and archaeal 16S rRNA genes, quantitative PCR (qPCR) reactions were performed in triplicate using an Applied Biosystems 7500 Real-time PCR system (Applied Biosystems, Waltham, MA, USA) with the SYBR green method (19). The abundance of bacterial and archaeal 16S rRNA genes was assessed using the *Bacteria*-specific primers 341F (5′-CCTACGGGAGGCAGCAG-3′) and 518R (5′-ATTACCGCGG CTGCTGG-3′) and the *Archaea*-specific primers A364aF (5′-CGG GGYGCASCAGGCGCGAA-3′) and A934b (5′-GTGCTCCCCCG CCAATTCCT-3′), respectively, as described in our previous study (19). The specificity of qPCR was confirmed by a melting curve analysis and gel electrophoresis.

### PCR amplification of bacterial and archaeal 16S rRNA genes and high-throughput sequencing

Triplicate PCR reactions were performed for each AS sample in a reaction volume of 25 μL, which consisted of 12.5 μL Failsafe Premix F (Epicentre Biotechnologies, Madison, WI, USA), 0.4 μM of each primer, 1 U of Platinum Taq DNA polymerase (Invitrogen, Carlsbad, CA, USA), and approximately 20 ng of a DNA template. The V1–V3 region of the bacterial 16S rRNA gene was amplified using the primer pair 27F (5′-AGAGTTTGATYMTGGCTCAG-3′) and 518R (5′-ATTACCGCGGCTGCTGG-3′) (20). Amplification was performed under the following conditions: an initial denaturation at 95°C for 5 min, followed by 25 cycles of 95°C for 30 s, 55°C for 30 s, and 72°C for 90 s, and a final extension at 72°C for 7 min.

The V6 region of the archaeal 16S rRNA gene was amplified using the primer pair 958F (5′-AATTGGANTCAACGCCGG-3′) and 1048R (5′-CGRCRGCCATGYACCWC-3′) from AS samples (19). Amplification was performed under the following conditions: an initial denaturation at 95°C for 5 min, followed by 34 cycles of 95°C for 30 s, 60°C for 45 s, and 72°C for 60 s, and a final extension at 72°C for 7 min.

PCR products were purified using the MinElute Gel Extraction Kit (Qiagen, Valencia, CA, USA). The concentrations of purified PCR products were assessed in duplicate using the Quant-iT dsDNA HS assay kit (Molecular Probes, Sunnyvale, CA, USA). Bacterial and archaeal 16S rRNA gene amplicons were sequenced by 454 pyrosequencing and an Illumina HiSeq 2000 platform at Majorbio Bio-pharm Technology (Shanghai, China), respectively. The raw sequencing data of bacterial and archaeal 16S rRNA genes were deposited into the NCBI short reads archive database under accession numbers SRA049989 and SRX365129, respectively.

### Sequence analysis

Raw sequencing data were processed using QIIME v1.7.0 (5) and Mothur v1.3.1 (47) as described in our previous studies (18, 19). Briefly, for bacterial 16S pyrosequencing data, we excluded sequences that had a read length less than 150 bp, an average quality score less than 25, contained ambiguous bases (N), non-assigned barcodes, or mismatches in primer sequences, and contained a homopolymer run >6 bp. In order to avoid the overestimation of *α*-diversity, sequences were further denoised using Denoiser v0.91 (43). Regarding archaeal 16S Illumina HiSeq sequencing data, the barcoded Illumina PE sequencing pipeline was used to assemble raw paired-end reads into contigs (61). Contigs containing no ambiguous bases (N), without mismatches for barcodes or primers, and with lengths ranging between 50 and 90 bp were included in the downstream analysis. ‘pre.cluster’ and ‘chimera.uchime’ in Mothur v1.3.1 were used to correct potential sequencing errors and remove chimeras, respectively. The operational taxonomic units (OTUs) of bacterial and archaeal communities were identified with 97 and 98% identity cut-offs, respectively, using a uclust-based open-reference OTU picking pipeline (9). The taxonomic classification of bacterial and archaeal communities was performed using the RDP classifier with the Greengenes database v13_08 (32), at a 50% bootstrap cut-off value, and the results obtained were manually checked (19).

### Analysis of core-satellite and generalist-specialist taxa

Bacterial and archaeal OTUs were partitioned into core and satellite taxa using a chi-squared test of the index of dispersion (the ratio of variance to the mean abundance multiplied by occurrence) following the approach described in previous studies (20, 52). If the index of the dispersion of microbial OTUs follows a Poisson distribution (*i.e*., falls between the 2.5 and 97.5% confidence interval of the chi-squared distribution), these OTUs are randomly distributed (52). Habitat generalists and specialists were classified based on the occurrence of bacterial and archaeal OTUs with permutation algorithms (1,000 permutations) using EcolUtils (v0.1 package, https://github.com/GuillemSalazar/EcolUtils). This approach generated 1,000 stimulated OTU tables using the quasiswap algorithm. The occurrence of OTUs was calculated based on 1,000 randomly generated OTU tables, and the observed occurrences for the true microbial communities were then compared to the 95% confidence interval derived from the means of randomly generated occurrences. Habitat generalist and specialist OTUs were identified with observed occurrences that were higher than the upper 95% confidence interval and lower than the lower 95% confidence interval, respectively (58).

### Statistical analysis

The geographical distances between sample sites were converted from their geographic coordinates (longitude and latitude). Environmental similarities were detected using Ward’s linkage clustering with z-score-normalized environmental variables (except for the geographical distance). Prior to the analysis of high-throughput sequencing data, the read numbers of bacterial and archaeal communities were normalized to 7,000 and 12,000 reads per sample, respectively. The permutational multivariate analysis of variance (Adonis), analyses of similarity (ANOSIM), and the multi-response permutation procedure (MRPP) were used to test for significant variations among the key ecological groups (*i.e*., core-satellite and habitat generalist-specialist) of AS microbial communities between different locations (*i.e*., Chongqing versus Xiamen) (18, 31, 55). The principal coordinate analysis (PCoA) was used to display the *β*-diversity patterns of different ecological groups of microbial communities based on the Bray-Curtis dissimilarity matrix. The OTU table was standardized by the Hellinger transformation. A partial distance-based redundancy analysis (db-RDA) was performed to evaluate the relative contributions of environmental and spatial factors to microbial community structures with 9,999 permutations (19). We introduced spatial models by applying the method of the principal coordinates of neighborhood matrices (PCNM), and spatial descriptors were obtained by PCNM (4). Prior to db-RDA, highly auto-correlated environmental variables (Spearman’s correlation: *r*>0.70, *P*<0.05) were excluded (Fig. S4).

The abundance-based β-diversity null model developed by Tucher *et al*. (2016) was used to evaluate the relative importance of deterministic and stochastic processes on the community assembly of key microbial ecological groups in the 13 WWTPs examined (49). The null distribution of the expected *β*-diversity was generated for 999 randomly generated microbial communities. A *β*-null deviation was then calculated as the difference between the observed mean *β*-diversity and expected mean *β*-diversity (49). All statistical analyses were performed using R v3.20 with the packages phyloseq (33), ggplot2 (56), ecodist (13), vegan (v1.15-2 package, http://CRAN.R-project.org/package=vegan), and custom R scripts.

## Results

### Environmental characteristics of AS samples

The characteristics of AS samples from WWTPs and environmental parameters are summarized in Tables S1 and S2, respectively, in the supplemental materials. Eight out of the 13 WWTPs are located in Chongqing. The average distances among these WWTPs are 94.35 km, ranging between 35.06 and 175.05 km. The average distances among the remaining five WWTPs in Xiamen are 11.95 km, ranging between 5.42 and 17.04 km. The average distances between WWTPs from Chongqing and Xiamen are 1,205.82 km, ranging between 1,150.84 and 1,280.09 km (Fig. S1 and S2A).

Based on the concentrations of heavy metals, we identified three groups: a) X5 was distinct from all other WWTPs because Cr, Ni, Cu, Zn, and Pb concentrations were significantly higher in these samples (Table S2); b) samples collected from Chongqing were grouped together with the X3 sample; and c) the remaining samples (X1, X2, and X4) from Xiamen formed a unique group (Fig. S2B). Cluster analyses based on physicochemical variables (pH, NH_4_-N, NO_2_-N, NO_3_-N, dissolved inorganic nitrogen (DIN), OrgC, OrgN, OrgS, and OrgC/OrgN) revealed slightly different clustering patterns. Three AS samples from Xiamen (X1, X2, and X5) were grouped together, while samples from Chongqing and those from the remaining two WWTPs from Xiamen formed another group (X3 and X4) (Fig. S2C). Cluster profiling of all environmental parameters showed a similar classification for heavy metals (Mantel test, *r*=0.52, *P*=0.02): the higher heavy metal concentrations of AS sample X5 were distinct from the others, and C7 was grouped with X1, X2, and X4 (Fig. S2D). The Mantel test indicated that the clustering profile of heavy metals was related to geographical distance (*r*=0.39, *P*=0.002), while the correlation between physicochemical variables and geographical distance was relatively weak (*r*=0.34, *P*<0.05).

### Abundance of bacterial and archaeal 16S rRNA genes

qPCR results indicated that the abundance of the bacterial 16S rRNA gene in the AS samples of WWTPs from Xiamen and Chongqing ranged between 8.12×10^6^ and 5.48×10^10^ copies ng DNA^−1^, while the abundance of the archaeal 16S rRNA gene varied between 2.91×10^3^ and 2.38×10^7^ copies ng DNA^−1^ (Table S2), which were significantly lower than those of *Bacteria* (Wilcoxon test, *P*<0.001). The ratio of bacterial to archaeal 16S rRNA genes ranged between 93.6 and 3.81×10^4^, which was consistent with the findings of a previous study on six industrial and domestic WWTPs (2). Moreover, a positive correlation was observed between the abundances of bacterial and archaeal 16S rRNA genes in the AS samples investigated (Spearman’s correlation: *r*=0.81, *P*<0.001).

### OTU analysis of ecological groups

A total of 91,000 bacterial and 156,000 archaeal 16S rRNA gene sequences were included in further analyses after sequence processing. A total of 5,457 and 4,055 OTUs were obtained for bacterial and archaeal communities, respectively. The rarefaction analysis indicated that the sequencing depth was still insufficient to capture the full richness of AS microbial communities from the WWTPs of Chongqing and Xiamen (Fig. S3), and, thus, further sequencing may be valuable for identifying more key ecological species. Among the bacterial OTU data set, 1,388 core OTUs (25.4% of total OTUs), 1,038 satellite OTUs (19.0%), 255 habitat generalist OTUs (4.7%), and 192 habitat specialist OTUs (3.5%) were identified (Table S3). Regarding *Archaea*, 369 core OTUs (9.1%), 1,683 satellite OTUs (41.5%), 48 habitat generalist OTUs (1.2%), and 111 habitat specialist OTUs (2.7%) were obtained (Table S3). The proportion of core OTUs among total OTUs was higher than that of habitat satellite OTUs. The pattern for the habitat generalist OTUs identified was similar to that for the habitat specialist OTUs within bacterial communities. However, ecological groups showed an opposite pattern for archaeal OTUs (Table S3).

The reads of key ecological groups presented variable patterns. Overall, within *Bacteria* and *Archaea*, core OTUs were more abundant than habitat specialists. Specifically, for *Bacteria*, core OTUs with 25.4% of total OTUs accounted for 84.4% of total reads, and archaeal core OTUs constituted only 9.1% of total OTUs, but accounted for 88.4% of total reads. In contrast, satellite OTUs comprised more than 19.0% of all bacterial OTUs, but only accounted for a small proportion of total reads (5.6%). Archaeal satellite OTUs exhibited a similar pattern, suggesting that the satellite species in AS are highly diverse. Habitat generalists and specialists both occupied a small proportion of total reads. In *Bacteria*, habitat specialists were more abundant, whereas habitat generalists represented higher diversity.

**Fig. 1:**
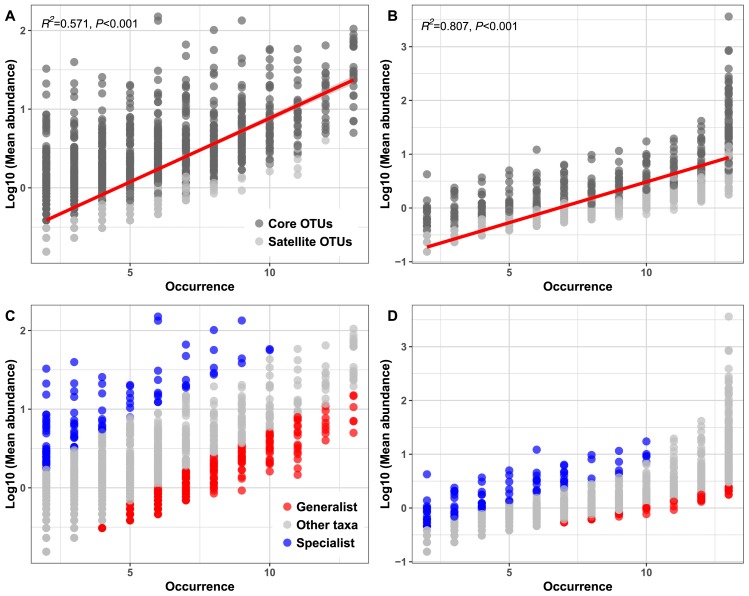
Abundance-occupancy relationship of bacterial (A, C) and archaeal (B, D) taxa in 13 WWTPs. In Fig. 1A and B, dark grey and grey colors represent core and satellite taxa, respectively, and the red lines represent the linear regression model fit to the species abundance distribution. The coefficients of linear models were shown in Fig. 1A–D. In Fig. 1C and D, red and blue colors represent generalist and specialist taxa, respectively.

A positive correlation was found between the mean abundance of OTUs and their occurrence (*Archaea*: *R^2^*=0.807, *P*<0.001; *Bacteria*: *R^2^*=0.571, *P*<0.001) (Fig. 1A and B), which was consistent with previous findings (50). The abundance-occupancy relationship of habitat specialists and generalists is shown in Fig. 1C and D, demonstrating that habitat generalists are more widespread. Although larger numbers of habitat generalists were detected in most of the WWTPs, habitat specialists were only abundant in certain niches.

**Fig. 2:**
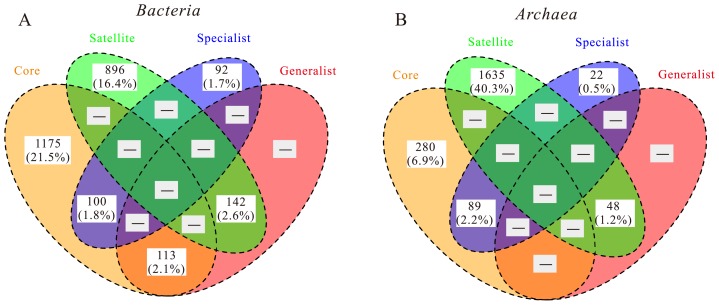
Venn diagram showing the number of bacterial (A) and archaeal (B) OTUs shared among four ecological groups (*i.e*. core, satellite, generalist, and specialist). Values in brackets represent the percentages of total OTUs.

The Venn diagram in Fig. 2 shows the number of unique and shared OTUs of the key ecological groups. Among bacterial OTUs, core and habitat generalist groups shared 113 OTUs (8.1% of total core OTUs), while core and habitat specialist communities shared 100 OTUs (7.2% of total core OTUs). A total of 142 bacterial OTUs (13.6% of total satellite OTUs) were shared between satellite and habitat generalist groups, while no OTUs were shared between satellite and habitat specialist groups. Within *Archaea*, none of the OTUs were commonly present between core and habitat generalist groups. However, the core and specialist groups of *Archaea* shared 89 OTUs (31.8% of total core OTUs).

### Community compositions of key ecological groups of the AS microbiome

**Fig. 3:**
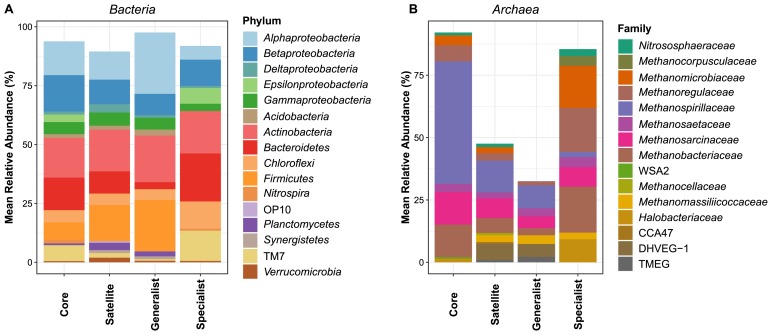
Taxonomic compositions of bacterial (A) and archaeal (B) taxa in core, satellite, habitat generalist, and habitat specialist groups. Only phyla or classes belonging to *Proteobacteria* accounting for more than 0.5% of the total sequences in each ecological group were shown for *Bacteria*. Only families accounting for more than 0.5% of the total sequences in each ecological group were shown for *Archaea*.

Significant differences were observed among the community compositions of core, satellite, habitat generalist, and habitat specialist groups in *Bacteria* and *Archaea* (Fig. 3). *Alphaproteobacteria*, *Betaproteobacteria*, *Actinobacteria*, *Bacteroidetes*, and *Chloroflexi* were the five most abundant phyla of the four bacterial ecological groups. Within *Bacteria*, the core group was dominated by *Actinobacteria* (16.8% of the total community), followed by *Betaproteobacteria* (15.5%) and *Alphaproteobacteria* (14.3%), while *Actinobacteria*, *Firmicutes*, and *Alphaproteobacteria* represented 17.7, 15.4, and 11.9%, respectively, of total abundance in the satellite group. *Deltaproteobacteria*, *Planctomycetes*, and *Verrucomicrobia* with relatively lower abundances in the core and satellite groups constituted a major proportion of habitat specialists. The taxonomic composition of archaeal communities mainly encompassed 15 families (Fig. 3B), with most sequences being affiliated with *Methanosarcinacea*, *Methanobacteriaceae*, and *Methanospirillaceae*. Relative abundances at a major taxonomical level were significantly different for the four ecological groups. *Methanospirillaceae*, *Methanosarcinaceae*, and *Methanobacteriaceae* were prevalent in core and satellite groups. However, these families were present at a lower proportion in archaeal habitat generalists, which were the most divergent from the other groups. Archaeal habitat specialists were mainly dominated by *Methanobacteriaceae*, *Methanoregulaceae*, and *Methanomicrobiaceae* (18.4, 17.7, and 16.9%, respectively, of the total community) (Fig. 3B). In addition, *Nitrososphaeraceaea* constituted a higher proportion in habitat specialists (2.8%) than in habitat generalists (<0.5%) (Fig. 3B).

### Community structure of key ecological groups of the AS microbiome

**Fig. 4:**
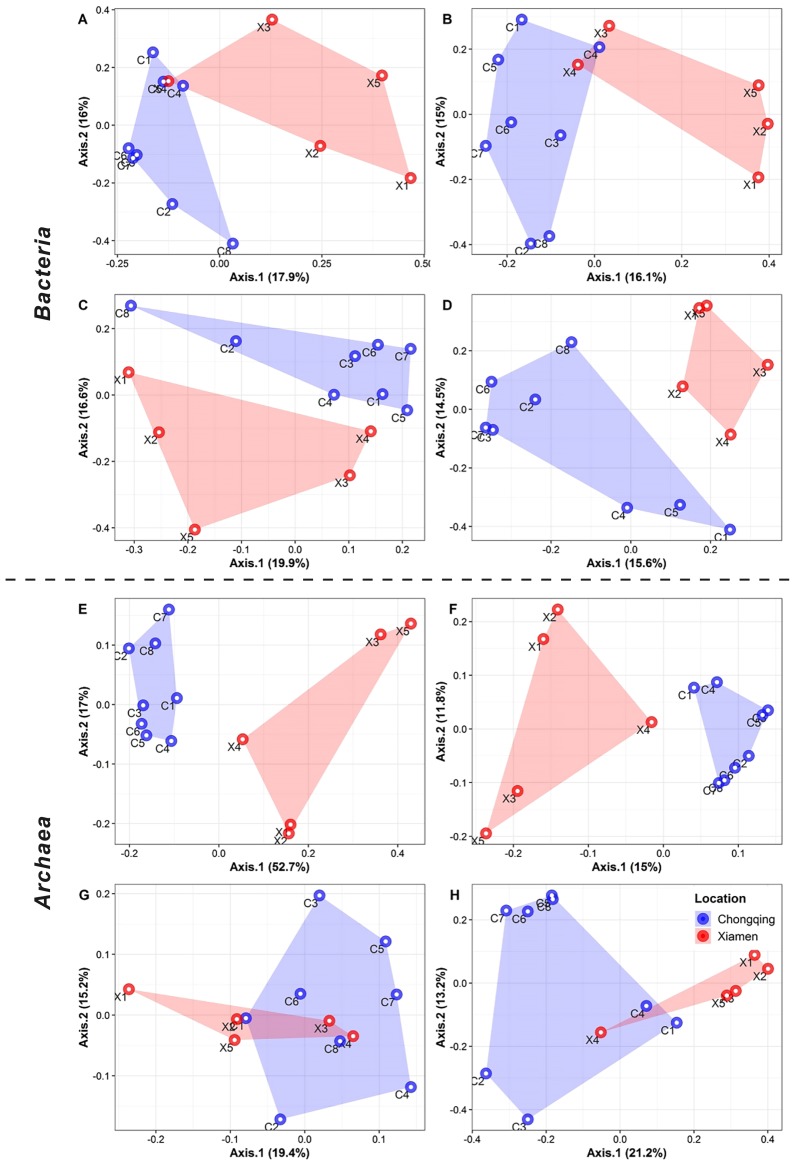
PCoA ordination with the Bray-Curtis dissimilarity matrix of four ecological groups of bacterial (A–D) and archaeal (E–H) communities in WWTP. Fig. 4A and E, B and F, C and G, and D and H represent core, satellite, habitat generalist and habitat specialist groups, respectively.

Adonis, ANOSIM, and MRPP were applied to evaluate the significance of differences between two locations (*i.e*., Chongqing versus Xiamen) for each ecological group (Table 1). The results of Adonis tests showed similarities between the two locations for the four bacterial ecological groups (core: *R^2^*=0.15; satellite: *R^2^*=0.14; habitat generalist: *R^2^*=0.15; and habitat specialist: *R^2^*=0.15), while the *R^2^* value of the core group (core: *R^2^*=0.44) was markedly higher than those of the other archaeal groups (satellite: *R^2^*=0.14; habitat generalist: *R^2^*=0.11; and habitat specialist: *R^2^*=0.16). However, the grouping of habitat generalists from two cities was not significant (*P*=0.094) in *Archaea*. The results of ANOSIM and MRPP analyses were consistent with those of Adonis tests. According to PCoA based on the Bray-Curtis dissimilarity matrix, sample pattern clustering by locations confirmed the results of Adonis, ANOSIM, and MRPP tests (Fig. 4). This approach revealed a clear distinction among the four ecological groups found in the two locations for the bacterial communities (Fig. 4A, B, C, and D). Within *Archaea*, core and satellite groups from different locations clustered apart, while some samples of habitat generalists and specialists from the WWTPs in Xiamen segregated closer to the WWTPs in Chongqing (Fig. 4E, F, G, and H).

### Relationship between environmental factors and community structures of AS ecological groups in *Bacteria* and *Archaea*

db-RDA revealed variations in the relative importance of environmental and spatial factors (*i.e*., PCNM) responsible for explaining variations in the community compositions of four ecological groups in two domains (Table 2). The best predictors differed for the bacterial and archaeal communities. NH_4_-N, OrgC/OrgN, Cr, and Ni were the variables that best explained variations in the bacterial core community composition, while Cr, Ni, and As were the best for the archaeal core community. Ag was the only significant predictor common to both bacterial habitat generalists and specialists. Within *Bacteria*, four ecological groups were related to PCNM 1, 7, and 8, with PCNM 1 being important for all groups. PCNM 1, 5, and 9 were selected as a significant variable for archaeal ecological groups. However, no PCNM variables were related to archaeal habitat generalists. The relative contributions of environmental and spatial factors to community compositions were tested (Table 2). In core communities, environmental factors were explained with a significantly higher percentage for *Bacteria* (17.4%, *P*<0.001) than for *Archaea* (10.7%, *P*<0.001). Variances in spatial factors (*Bacteria*: 5.8%, *P*<0.01; *Archaea*: 2.6%, *P*<0.01) were slightly higher than those in environmental factors (*Bacteria*: 5.3%; *Archaea*: 0.1%) for the satellite community. Variances in environmental factors were lower than those in spatial factors for bacterial habitat generalists. However, habitat generalists and specialists were not explained well by the measured environmental and spatial variables through partial db-RDA.

### Detecting underlying assembly mechanisms using null models of β-diversity

**Fig. 5:**
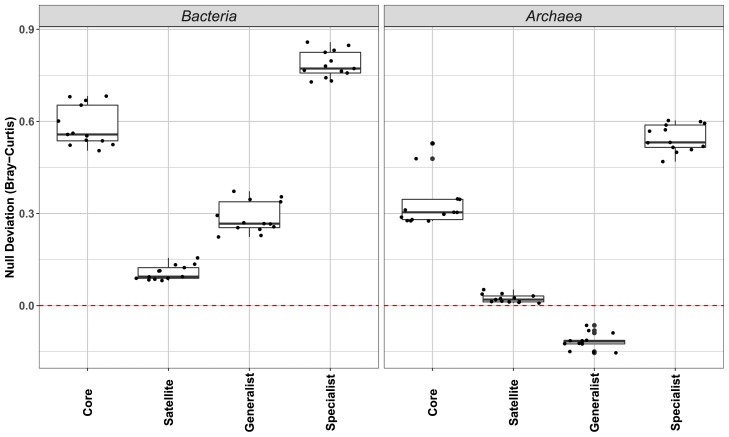
Relative importance of niche and neutral processes, which were assessed using deviations from abundance-based *β*-null models (Bray-Curtis dissimilarity), on the assembly of different ecological groups of bacterial and archaeal communities in 13 WWTPs.

Deviations in abundance-based *β*-null models (Bray-Curtis dissimilarity) were used as an indicator of the ecological processes structuring communities. Deviations from zero in *β*-null deviation values indicated an increase in the relative importance of deterministic processes on community assembly, and values near to zero reflected assembly dynamics that were slightly stochastic (49). The present results clearly showed that the *β*-null deviation values of the core groups in *Bacteria* and *Archaea* were significantly higher than those of the satellite groups (Wilcoxon test, *P*<0.001), while generalists had significantly lower *β*-null deviation values than those of habitat specialists in the two domains (Wilcoxon test, *P*<0.001) (Fig. 5). In other words, the *β*-null deviation values of satellite and habitat generalist communities in AS were closer to zero than their counterparts (Fig. 5). This result indicated that the relative importance of deterministic processes was greater for the assembly of core and habitat specialist microbial communities than for the assembly of satellite and habitat generalist communities. Similar results were also obtained based on an analysis of 46 AS samples, which were collected from seven WWTPs in Xiamen in 2016 (Fig. S5).

## Discussion

A core community with abundant microorganisms may play a key role in maintaining the stability of the AS process by contributing to carbon turnover and nutrient removal (45). Previous studies demonstrated that core species were best adapted to the local environment due to their ability of utilize widespread resources, whereas satellite species had limited dispersal (3, 29, 50). Since the core taxa examined in the present study were dominant in all WWTPs investigated in the two cities (Table S3 and Fig. 1), it is highly likely that that these core taxa were mainly responsible for maintaining AS long-term functional stability (29). The present results revealed that *Actinobacteria*, *Betaproteobacteria*, and *Alphaproteobacteria* had higher abundances in the core than in the satellite communities, and the latter two classes were considered to be important functional groups for nutrient removal in AS (57, 59, 60). Similar core taxa were also detected in Danish conventional AS systems, confirming that bacterial core taxa are widespread in different AS, and that the driving force of core community formation is similar (15, 45). However, in a recent study on 20 full-scale WWTPs (39), the archaeal core community was dominated by *Methanosaeta*, *Methanosarcina*, *Methanogenium*, *and Methanobrevibacter*, which differs from the present results. Since *Methanosaeta* was previously reported to be the dominant archaeal taxa in the aerobic AS process (14), the predominance of *Methanosarcinaceae* in this study may have been due to the higher acetate concentration produced in anaerobic or anoxic zones during the AS process.

In contrast to previous findings (27), the present results showed that habitat specialists had a higher abundance in certain niches/sites. One possible explanation may be the application of a novel statistical method for identifying habitat generalists and specialists (58), which may avoid biases generated by other classification methods (30, 48). However, consistent with recent findings obtained using a different classification approach (27, 36), generalists in the present study harbored a large abundance of taxa belonging to *Proteobacteria* and *Actinobacteria*. These results indirectly prove the validity of our habitat generalist identification method. Another explanation may be that habitat specialists play a vital role and have special ecological functions in particular niches in AS (17). This speculation is somewhat supported by the present results, with a higher proportion of *Nitrososphaeraceaea* being identified in habitat specialists than in generalists, suggesting a greater contribution from ammonia-oxidizing archaea to remove nitrogen nutrients in some of the WWTPs investigated (10).

The present results revealed that core groups had similar or larger numbers of shared OTUs with habitat specialists (Fig. 2), implying a potential link between core and habitat specialist groups. This is inconsistent with the general assumption of a considerable overlap between the OTUs of core and habitat generalist groups based on their common ecological characteristics (20, 48, 52). This inconsistency may be explained by methodological differences between the present study and previous surveys (see the above discussion). Nevertheless, the overlap between core and habitat specialist groups suggests that habitat specialists play an important role in the AS process under certain conditions. For example, the top two dominant classes of habitat specialists—*Bacteroidetes* and *Actinobacteria*—, which were found to dominate in four full-scale WWTPs in Shanghai (11), accounted for a relatively higher proportion in the bacterial core community. In a survey of WWTPs with intermittent or continuous bulking and foaming issues, *Actinobacteria—*related taxa were often identified as the key microorganisms (47). Another dominant class of habitat specialists—TM7 (filamentous microbes)—represented the main trigger of bulking issues, but also made significant contributions to floc formation and the stability of WWTPs (38, 47). Collectively, these findings suggest that habitat specialists have significant effects on AS stability.

In a study on microbial communities in geographically distributed WWTPs, Xia *et al*. (59) reported that the commonality of the bacterial community was stronger in bioreactors within China than in those between China and United States. Another study found a clear geographic signal in bacterial communities among 14 WWTPs from four countries by using 16S-pyrotag (60). In the present study, the community structures of ecological groups significantly differed among the investigated cities (Table 1). However, for bacterial and archaeal core communities, variations in community compositions correlated more strongly with environmental factors than spatial factors, implying the essential role of deterministic processes in shaping the structure of core communities in 13 WWTPs in the two cities. Specifically, in accordance with a previous study, NH_4_-N, OrgC/OrgN, Cr, and Ni were the factors responsible for variations in the composition of the bacterial core community in AS (24). Furthermore, the present results also indicated that variations in the structures of satellite communities were better explained by spatial factors than by local environmental factors, implying that a higher degree of stochasticity is associated with the satellite community (Table 2).

Variations in the compositions of habitat generalist and specialist communities were generally not explained by environmental variables, presumably due to the effects of unmeasured environmental factors or dispersal limitations (27, 36). Although the composition of the bacterial habitat specialist community correlated with spatial factors, previous findings indicated that other environmental factors that were not measured in the present study (*e.g*., biochemical oxygen demand, COD, total suspended solids, temperature, DO, and conductivity) significantly influence the microbial community structure in full-scale WWTPs (24, 39, 51, 54). This may also explain the relatively low explanatory power of the partial db-RDA models for core communities.

The present results, which were based on abundance-based *β*-null models (Fig. 5), were consistent with previous findings showing that deterministic and stochastic processes played different roles in driving the assembly of different key ecological groups within microbial communities (27). Moreover, the relative importance of deterministic and stochastic processes varied for different phyla/classes in natural environments (*e.g*., sea, freshwater, soils, and sediments) (22, 29, 48, 58, 63). However, the assembly mechanisms of the key ecological groups within AS microbial communities have not yet been elucidated in detail (34). Ofiteru *et al*. previously reported that a WWTP microbial community assembly was dependent on stochastic processes caused by chance and random immigration (40). On the other hand, a recent study showed that seasonal changes in the core community composition in bio-reactors were governed by deterministic assembly due to seasonal temperature fluctuations, while the assembly dynamics of rare taxa were slightly stochastic (15). The present results obtained using abundance-based *β*-null models support this finding by showing that stochastic and deterministic processes shaped the assembly of satellite (*β*-null deviation values near zero) and core communities (values further away from zero) of *Bacteria* and *Archaea*, respectively, in AS from 13 WWTPs (Fig. 5). This is also consistent with the results obtained using partial db-RDA models, suggesting that spatial variations in AS core communities are caused by changes in environmental conditions among WWTPs. Similar findings have been reported in other environments. For example, in a study on microbial communities in coastal sediments, core bacterial, archaeal, and eukaryotic communities showed the highest sensitivities to changes in environmental and contaminant conditions (22).

In contrast to the partial db-RDA model, the abundance-based *β*-null model does not rely on external factors (*i.e*., spatial and environmental factors) to assess the relative importance of different ecological processes (49). Therefore, this model may contribute to a better understanding of the assembly mechanisms of habitat generalist and specialist communities; however, the explanatory power of partial db-RDA models is low for spatial and environmental factors. The assembly dynamics of habitat generalists (values near zero) and specialists (values further away from zero) were mainly shaped by stochastic and deterministic processes, respectively, based on the degrees of deviation in *β*-null deviation values from zero. Similarly, in a survey on microbial habitat generalists and specialists in 21 plateau lakes of China, habitat specialists were mainly shaped by deterministic factors. Furthermore, a similar assembly mechanism (deterministic processes) governing core and habitat specialist communities may be due to a high proportion of shared OTUs between them.

In summary, the assembly mechanisms structuring the distribution of the key ecological groups within two domains (*i.e*., *Bacteria* and *Archaea*) were studied in the AS microbiome from 13 municipal WWTPs in two cities in China. Consistent with our second and third hypotheses, we found that (i) the assembly of core and satellite communities was mainly shaped by deterministic and stochastic processes, respectively; and (ii) stochastic processes strongly influenced habitat generalist communities, while deterministic processes dominated the assembly of habitat specialist communities. However, core and habitat specialist communities had a considerable overlap, suggesting that habitat specialists play a similar role in AS process as the core group under certain conditions. The results of the present study provide valuable insights into the underlying ecological processes that govern the assembly of AS microbial communities at the population (*i.e*., key ecological groups) level rather than at the community level. However, further studies based on sequencing in more depth and larger sample sizes from multiple sites/cities are required to improve our understanding of the assembly dynamics of key microbial ecological groups in the AS process. Additionally, more detailed information on environmental or operational parameters needs to be included in future studies for the better identification of the main determining factors driving the distribution of key ecological groups, particularly core and habitat specialist communities.

## Supplemental Materials



## Figures and Tables

**Table 1:** Significance tests on community structures of four ecological groups of *Bacteria* and *Archaea* between locations (*i.e*., Chongqing versus Xiamen) with Adonis, ANOSIM, and MRPP tests.

Domain	Ecological groups	Adonis			ANOSIM		MRPP	
		
		*F*	*R^2^*	*P*	*R*	*P*	*δ*	*P*
*Bacteria*	Core	**1.94**	**0.15**	**0.002**	**0.43**	**0.004**	**0.04**	**0.005**
	Satellite	**1.75**	**0.14**	**0.002**	**0.52**	**0.003**	**0.03**	**0.005**
	Habitat Generalist	**1.87**	**0.15**	**0.006**	**0.42**	**0.006**	**0.03**	**0.011**
	Habitat Specialist	**1.78**	**0.14**	**0.003**	**0.51**	**<0.001**	**0.03**	**0.003**

*Archaea*	Core	**8.69**	**0.44**	**0.001**	**0.79**	**0.001**	**0.21**	**0.001**
	Satellite	**1.77**	**0.14**	**0.001**	**0.73**	**<0.001**	**0.03**	**0.002**
	Habitat Generalist	1.39	0.11	0.094	0.17	0.09	0.02	0.097
	Habitat Specialist	**2.03**	**0.16**	**0.006**	**0.3**	**0.02**	**0.05**	**0.006**

**Table 2:** Results of db-RDA models.

Domain	Method		Ecological groups			

			Core	Satellite	Generalist	Specialist
*Bacteria*	Partial db-RDA	Selected Envs^a^	2, 6, 7, 8	2, 6	12	12
		Selected Spat^b^	PCNM1	PCNM1, PCNM7	PCNM1, PCNM8	PCNM1, PCNM8
		Pure Env^c^	0.174**^d^	0.053	0.000	0.004
		Pure Spat	0.000	0.058*	0.068	0.075*
		shared	0.109	0.046	0.085	0.085

*Archaea*	Partial db-RDA	Selected factors	7, 8, 9	5	NA	NA
		Selected Spat	PCNM1	PCNM1	NA	PCNM5, PCNM9
		pure Env	0.107**	0.001	NA	NA
		pure Spat	0.05*	0.026*	NA	0.105
		shared	0.204	0.035	NA	NA

a Envs: 1. pH; 2. NH_4_-N; 3. NO_2_-N; 4. NO_3_-N; 5. OrgS; 6. OrgC/OrgN; 7. Cr; 8. Ni; 9. As; 10. Cd; 11. Pb; 12. Ag.

b Selected Spat: a principal coordinate of neighborhood matrices (PCNM) variables

c Pure Env, pure effect of environmental factors; Pure Spat, pure effect of geographic distance; shared, shared effect between environmental and geographic factors.

d The explained variance (*R_2_*) of the db-RDA model was reported based on 9,999 permutation tests.

NA, not available,

*** *P*≤0.001,

** *P*≤0.01,

* *P*≤0.05.
