# Supplementary material for: Deciphering the Assembly Processes of the Key Ecological Assemblages of Microbial Communities in Thirteen Full-Scale Wastewater Treatment Plants
Source: Microbes Environ. 2019 Apr 16;34(2):169–79. doi: 10.1264/jsme2.ME18107 (PMC6594736; doi:10.1264/jsme2.ME18107)
Supplement: Supplementary file 1 [file 34_169_s1.pdf]

Supplemental Materials

Decipher the assembly processes of the key ecological assemblages of microbial communities in thirteen full-scale wastewater treatment plants

Running title: Assembly of WWTP Microbial Assemblages

Liyuan Hou[1,2], Anyi Hu[1*], Shaohua Chen[1],

Kaisong Zhang[1], Sandi Orlić[3,4], Azhar Rashid[1,5], Chang-Ping Yu[1,6]

1. CAS Key Laboratory of Urban Pollutant Conversion, Institute of Urban Environment, Chinese Academy of Sciences, Xiamen 361021, P. R. China
2. Department of Civil and Environmental Engineering, University of Missouri, USA
3. Ruđer Bošković Institute, Bijeničkacesta 54, 10000 Zagreb, Croatia
4. Center of Excellence for Science and Technology–integration of Mediterranean region–STIM, Bijeničkacesta 54, 10000, Zagreb, Croatia
5. Nuclear Institute for Food and Agriculture, Tarnab, Peshawar, Pakistan.
6. Graduate Institute of Environmental Engineering, National Taiwan University, Taipei 106, Taiwan

***Correspondence to:**

Dr. Anyi Hu, ayhu@iue.ac.cn

CAS Key Laboratory of Urban Pollutant Conversion, Institute of Urban Environment, Chinese Academy of Sciences, Xiamen 361021, P. R. China, Telephone & Fax: 86-592-6190582.

Table S1 Characteristics of 13 WWTPs located in Chongqing and Xiamen, China.

City	WWTPs	Core Treatment processes	Population served	Average flow (10^4 m^3/day)	Average sludge production (kgDS/day or m^3/day)[a]	HRT (h)	SRT (d)
Chongqing	**C1**	Modified Orbal oxidation ditch	11.71	3.3	4186	19.8	25.3
	**C2**	Modified Carrousel oxidation ditch	21.46	3.9	8507	7.6	5.8
	**C3**	Modified oxidation ditch	5.92	1.4	3847	15.1	10.6
	**C4**	Modified oxidation ditch	14.43	3	3655	14.2	18.8
	**C5**	Modified oxidation ditch	15.05	1.6	3178	10.1	6
	**C6**	SBR	16.01	1.4	1962	35.3	31.9
	**C7**	Modified oxidation ditch	8.83	1.6	3663	9.5	7.3
	**C8**	Oxidation ditch	17	2.5	5501	15.1	10.2
Xiamen	**X1**	BAF	28	24.5	14.8	NA	NA
	**X2**	A/O	48.16	10.1	37	NA	NA
	**X3**	Modified oxidation ditch	14.3	4.7	49	NA	NA
	**X4**	Orbal oxidation ditch	17.15	3.9	13	NA	NA
	**X5**	A^2/O	20	5.1	48	NA	NA

Abbreviations: SBR: sequencing batch reactor; BAF: biological aerated filter; A/O: anoxic/aerobic; A^2/O: anaerobic/anoxic/aerobic; NA: not available.

[a]The sludges were produced in the unit of kgDS per day for WWTPs in Chongqing and m^3 per day (water content about 76%) for WWTPs in Xiamen.

Table S2. The environmental parameters and the abundance of bacterial and archaeal 16S rRNA genes of 13 AS samples investigated in this study.

City	WWTPs	Cr (mg/kg)	Ni (mg/kg)	Cu (mg/kg)	Zn (mg/kg)	As (mg/kg)	Cd (mg/kg)	Pb (mg/kg)	Ag (mg/kg)	pH	NH_4-N (mg/kg)	NO_2-N (mg/kg)	NO_3-N (mg/kg)	DIN (mg/kg)[b]	OrgC (%)	OrgN (%)	OrgS (%)	OrgC /OrgN	Air temp (℃)[c]
Chongqing	**C1**	8.95	3.71	23.32	55.8	4.63	1.22	40.4	0.8	7.53	180.36	0.27	ND	180.63	25.31	3.91	1.68	6.48	11 ± 5
	**C2**	8.23	3.5	15.55	55.7	4.37	1.1	25.0	0.89	7.87	482.48	0.07	0.21	482.76	26.53	4.42	2.58	6.0	11 ± 5
	**C3**	7.0	3.88	13	51.85	4.26	1.1	17.02	0.73	6.17	78.33	0.26	2.23	80.82	31.44	5.37	1.42	5.86	11 ± 5
	**C4**	19.86	3.65	16.31	63.75	7.6	0.95	22.71	1.03	7.83	339.07	0.09	ND	339.16	29.59	5.15	1.52	5.74	11 ± 5
	**C5**	7.52	3.99	19.08	57.68	3.65	1.51	29.78	1.4	7.62	183.46	0.32	0.4	184.18	26.63	4.82	1.19	5.52	11 ± 5
	**C6**	16.57	3.89	13.55	61.35	4.99	1.47	32.35	0.66	7.74	223.27	0.56	ND	223.83	26.83	5.0	1.34	5.37	11 ± 5
	**C7**	5.34	2.87	7.48	26.0	3.12	0.98	17.15	0.57	7.85	383.29	0.01	0.27	383.57	18.23	3.5	3.85	5.2	11 ± 5
	**C8**	8.67	3.68	22.12	53.63	4.26	1.18	38.15	0.69	7.59	435.23	0.75	0.92	436.9	25.02	3.8	2.2	6.59	11 ± 5
Xiamen	**X1**	23.23	18.15	25.7	61.78	2.72	0.99	20.69	5.79	9.14	118.14	0.51	0.89	119.54	24.8	2.72	9.01	9.12	15 ± 6
	**X2**	27.35	19.98	73.48	59.98	3.22	0.43	30.68	1.28	9.16	217.42	0.22	0.97	218.61	15.62	2.19	5.21	7.12	15 ± 6
	**X3**	45.48	30.0	52.9	104.75	20.62	1.18	22.02	1.34	7.96	62.52	0.1	0.22	62.84	25.97	4.7	3.24	5.52	15 ± 6
	**X4**	47.8	22.37	57.83	157.4	3.41	0.69	21.57	3.11	7.51	201.14	ND[a]	ND	201.14	21.59	4.26	3.35	5.07	15 ± 6
	**X5**	1715.25	483.5	726	181.78	2.28	0.8	64.6	3.12	8.76	90.36	5.23	1.71	97.3	16.32	2.36	4.44	6.91	15 ± 6

[a]ND, not detected.

[b]Dissolved inorganic nitrogen (DIN) is the sum of NH_4-N, NO_2-N and NO_3-N.

[c]Air temp: the mean monthly air temperature during the sampling time was obtained from the meteorological agencies of Chongqing and Xiamen.

Table S2. Continued.

City	WWTPs	Bacterial 16S rRNA gene (copies/ng DNA)	Archaeal 16S rRNA gene (copies/ng DNA)	Ratio of Bacterial 16S rRNA gene/ Archaeal 16S rRNA gene
Chongqing	C1	1.27×10^9	6.85×10^5	1.86×10^3
	C2	3.38×10^7	1.15×10^5	2.93×10^2
	C3	9.36×10^8	3.19×10^4	2.94×10^4
	C4	1.20×10^9	3.83×10^6	3.14×10^2
	C5	7.67×10^8	1.27×10^6	6.04×10^2
	C6	1.22×10^8	3.21×10^3	3.81×10^4
	C7	4.52×10^8	1.21×10^5	3.73×10^3
	C8	8.12×10^6	2.91×10^3	2.79×10^3
Xiamen	X1	5.48×10^{10}	2.28×10^7	2.40×10^3
	X2	5.35×10^9	2.38×10^7	2.25×10^2
	X3	1.09×10^9	1.16×10^7	9.36×10^1
	X4	8.30×10^8	4.01×10^6	2.07×10^2
	X5	2.91×10^{10}	7.11×10^6	4.10×10^3

Table S3. The number of bacterial and archaeal OTUs and reads in each ecological groups, and their proportion in the total bacterial and archaeal communities in 13 WWTPs from Chongqing and Xiamen, China. The bacterial and archaeal communities were sub-sampled at the 7,000 and 12,000 reads per sample, respectively.

**Ecological groups**		**Bacteria**	**Archaea**
**Core**	Observed OTUs	1,388	369
	Percent in total OTUs	25.4%	9.1%
	Percent in total reads	84.8%	88.4%
**Satellite**	Observed OTUs	1,038	1,683
	Percent in total OTUs	19.0%	41.5%
	Percent in total reads	5.6%	10.2%
**Generalist**	Observed OTUs	255	48
	Percent in total OTUs	4.7%	1.2%
	Percent in total reads	6.8%	0.5%
**Specialist**	Observed OTUs	192	111
	Percent in total OTUs	3.5%	2.7%
	Percent in total reads	28.0%	2.3%

Fig. S1. Locations of Chongqing and Xiamen, China.

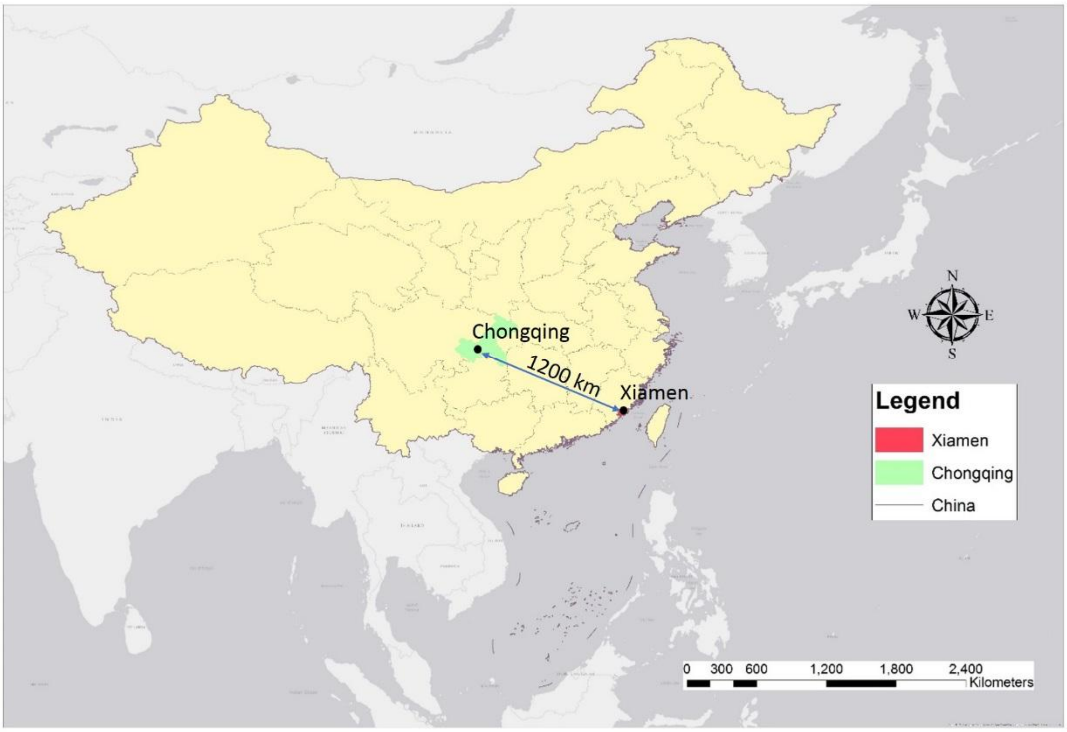

Fig. S2. Clustering of sampling sites based on geographic distance and complete linkage (A), and clustering of sampling sites based on heavy metals (Cr, Ni, Cu, Zn, As, Cd, Pb, and Ag) (B), physicochemical parameters (pH, NH_4-N, NO_2-N, NO_3-N, DIN, OrgC, OrgN, OrgC, OrgS, and OrgC/OrgN) (C), and all environmental variables of AS samples (D) based on Euclidean distance and ward linkage. Open circles indicate AS samples obtained from Xiamen and close circles indicate AS recovered from Chongqing. Scale bar indicates the geographic distance (A) or Euclidean distance (B, C and D), respectively.

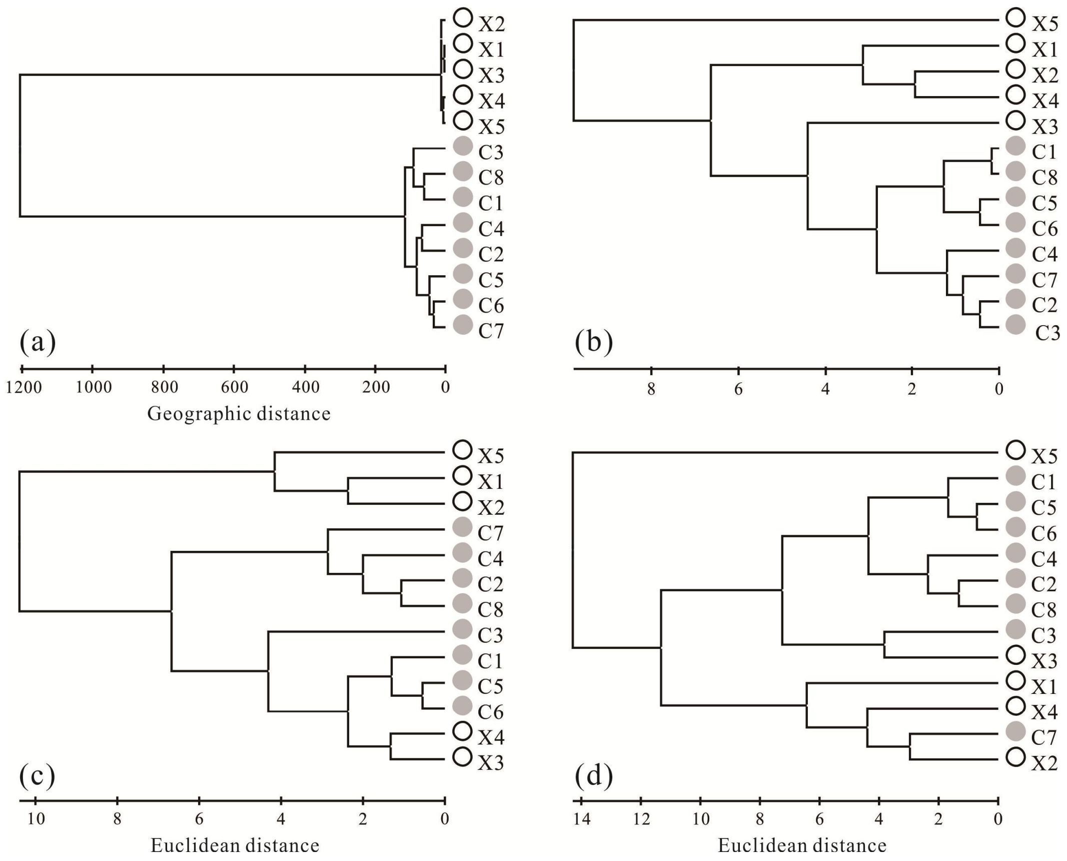

Fig. S3. Rarefaction curves of the observed OTU richness in AS bacterial (A) and archaeal (B) communities.

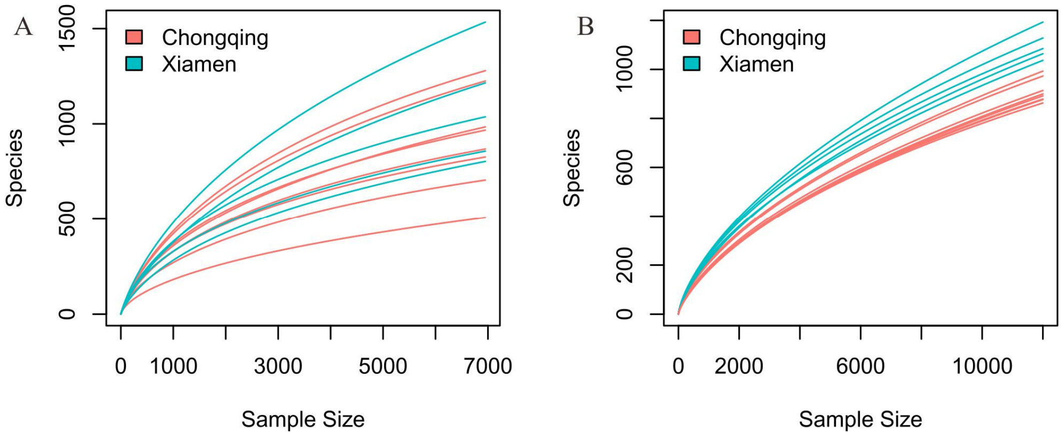

Fig. S4. Spearman correlation cluster analysis of the determined environmental parameters. The analysis was performed using 'varclus' function in Hmisc package.

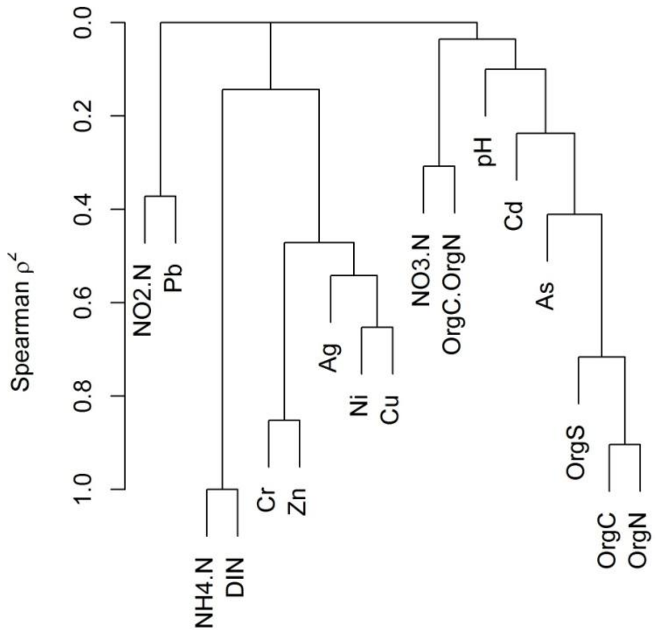

Fig. S5. The relative importance of niche and neutral processes, which were assessed using deviations from abundance-based β-null models (Bray-Curtis dissimilarity), on the assembly of different ecological groups of bacterial communities in 46 activated sludge samples collected from seven WWTPs in Xiamen during February 28th to March 4th, and March 6th, 2016[1].

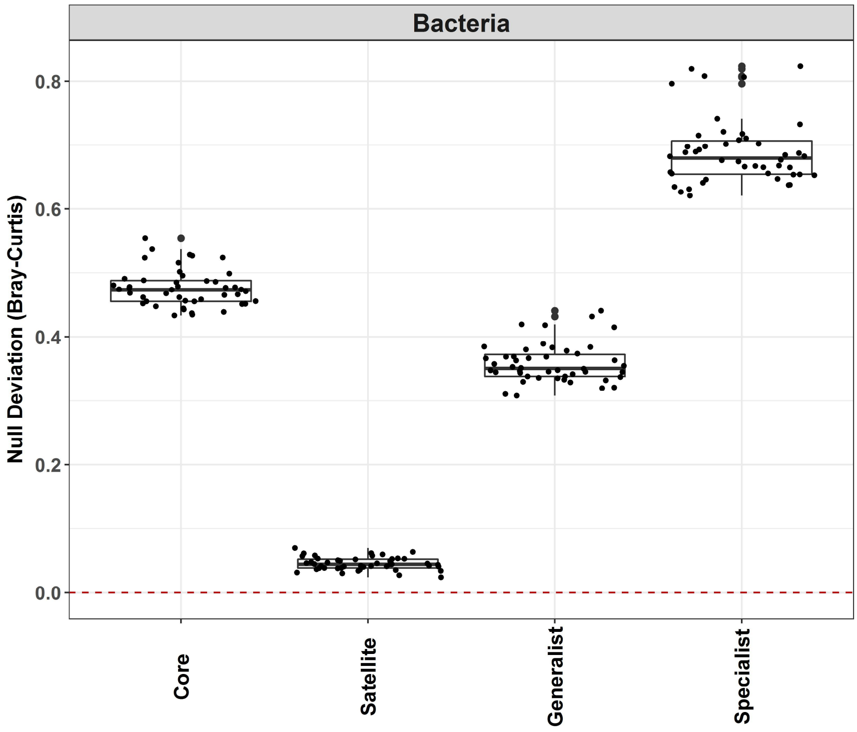

References

1. Sun Q, Wang Y, Li Y, Ashfaq M, Dai L, Xie X, Yu CP. Fate and mass balance of bisphenol analogues in wastewater treatment plants in Xiamen City, China. *Environ Pollut*. 2017, **225**: 542-549.
